# Evaluation of myocardial microcirculation changes in rats from different altitudes based on CT myocardial perfusion imaging

**DOI:** 10.1016/j.bbrep.2025.102417

**Published:** 2026-01-06

**Authors:** Chunlong Yan, Jinfeng Ma, Tingjun Yan, Yanqiu Sun

**Affiliations:** a Department of Radiology, Beijing Anzhen Hospital, Capital Medical University, Beijing Institute of Heart Lung and Blood Vessel Diseases, Beijing, China; b Department of Radiology, Jining No.1 People's Hospital, Jining, China; c Department of Radiology, Qinghai Provincial People's Hospital, Xining, China; d Department of Hematology, Jining No.1 People's Hospital, Jining, China; e Jining Medical College, Jining, China

**Keywords:** CT myocardial perfusion imaging, Myocardial microcirculation, Plateau, Hypoxia-inducible factor

## Abstract

This study employed computed tomography myocardial perfusion imaging (CT-MPI) to investigate myocardial microcirculation changes in rats across different altitudes. Four-week-old male Sprague-Dawley rats were allocated into plain (450 m), moderate-altitude (MA, 2200 m), and high-altitude (HA-A, 3800 m; HA-B, 4200 m) groups. After 28 weeks, CT-MPI revealed significant altitude-dependent alterations in myocardial perfusion parameters: TTP and MTT increased while BF and BV decreased (P < 0.05). Accompanying these changes were modifications in blood parameters—including red blood cell indices, white blood cell counts, and biochemical markers—along with myocardial structural remodeling characterized by disordered cell arrangement, widened intercellular gaps, and increased collagen deposition. Molecular analyses demonstrated variations in mRNA and protein expression of hypoxia-related markers (CD34, EPO, VEGF, HIF-1α, HIF-2α) and collagen types I/III, with HIF-2α exhibiting particular sensitivity to altitude variation. These findings indicate that high-altitude exposure induces progressive myocardial microcirculation impairment coupled with hematological adaptations and tissue remodeling, potentially mediated through HIF-2α-regulated hypoxia response pathways.

## Highlights

• Alterations in myocardial microcirculation in rats from different altitude regions.
• With increasing altitude, rat hearts exhibit a hypoperfusion state.
• We hypothesize that HIF-2α exhibits greater sensitivity to changes in altitude.

## 1 Introduction

High-altitude regions have unique geographic characteristics, including low pressure, low oxygen, strong ultraviolet radiation, dryness, and large temperature differences between day and night [1,2]. Among these, low pressure and low oxygen are the most prominent features [3]. When entering a plateau environment rapidly, with the increase in altitude, the oxygen content and partial pressure of oxygen in the atmosphere decrease. Initially, the human body compensates to adapt to the environmental changes. However, as the oxygen partial pressure in the alveoli decreases, the oxygen diffusing into the pulmonary capillary blood decreases as well, leading to a reduction in arterial oxygen partial pressure and saturation. When blood oxygen saturation drops to a certain level, it results in insufficient oxygen supply to organs and tissues, leading to symptoms such as fatigue, headache, dizziness, palpitations, shortness of breath, and insomnia. These symptoms represent the plateau response, which can develop into acute or chronic high-altitude diseases [4,5]. The cardiovascular system is particularly sensitive to hypoxia, which can result in structural and functional abnormalities in the heart [6], although the disease mechanisms are not yet fully understood.

As is well known, cardiovascular diseases have a high incidence rate in China, becoming one of the major health hazards to the population. Coronary angiography (CAG) is the “gold standard” for diagnosing coronary artery stenosis, but it only shows large epicardial coronary vessels, while many smaller arterioles and microvessels are not visible [7,8]. Severe coronary artery anatomical stenosis does not always lead to myocardial ischemia [9]. In recent years, myocardial microcirculation has become a hot topic. Some researchers have performed CAG on patients with myocardial ischemia symptoms and found that 67 % of patients with normal or near-normal CAG showed coronary epicardial vessel and microvascular spasm, indicating myocardial microcirculation dysfunction [[10], [11], [12]]. The mechanisms behind myocardial microcirculation disorders remain unclear. Many scholars believe that changes in myocardial microcirculation are influenced by multiple factors, such as metabolism, hormones, autonomic nervous system changes, and endothelial regulation [[13], [14], [15], [16]]. Studies have reported that, compared to patients without coronary microvascular dysfunction, those with coronary microvascular dysfunction (CMD) have an increased mortality rate and a higher risk of severe cardiovascular events [17]. In 2017, Chinese scholars published the first domestic and international expert consensus on the diagnosis and treatment of coronary microvascular disease (CMVD), which drew significant attention from the cardiovascular academic community [18]. More and more clinical doctors are focusing on the perfusion of myocardial microcirculation.

Currently, there is no direct imaging technique for assessing coronary microcirculation. Most assessments reflect microcirculatory conditions indirectly by evaluating microvascular function [19,20]. Non-invasive imaging methods for assessing myocardial microcirculation include transthoracic Doppler echocardiography (TTDE), myocardial contrast echocardiography (MCE), computed tomography (CT), positron emission tomography (PET), single photon emission computed tomography (SPECT), and cardiac magnetic resonance imaging (CMRI). Each method has its advantages. In recent years, with continuous advancements in CT hardware and software, CT myocardial perfusion imaging (CT-MPI) has evolved from traditional electron beam CT to dual-source dual-energy CT, with detector widths expanding from 16 slices to 320 slices, and image time and spatial resolution constantly improving while reducing radiation doses, further enhancing the maturity of CT-MPI technology [21]. Coronary computed tomography angiography (CCTA) is a fast and reliable tool for assessing coronary artery diseases, with high sensitivity and good negative predictive value, but it cannot estimate the hemodynamic significance of lesions [22]. CT-MPI technology, however, can accurately evaluate both coronary anatomy and myocardial perfusion, providing a combined anatomical and functional assessment, making it highly valuable for clinical applications [[23], [24], [25], [26]]. In the event of intravenous injection of contrast agent, CT myocardial perfusion imaging represents a continuous and fixed time-interval dynamic scan of selected slices. The time to peak (TTP), mean transit time (MTT), blood flow (BF), blood volume (BV), and other blood perfusion parameters of tissues and organs are utilized to quantitatively assess tissue microcirculation perfusion. These parameters can be employed to visualize changes in perfusion [27,28].

Previous studies on plateau diseases have mostly focused on acute mountain sickness, often using simulated environments like hypoxic chambers, which do not objectively reflect the physiological and pathological changes under real plateau conditions. Some scholars have analyzed the sleep quality of chronic mountain sickness(CMS) patients at different altitudes and found that sleep quality deteriorates as altitude increases, suggesting the need for proper sleep quality management in CMS patients in high-altitude areas. Our preliminary CT myocardial perfusion imaging studies have shown that patients with chronic hypoxia-induced polycythemia at high altitudes are in a state of low perfusion in the heart, and in chronic hypoxia environments, SD rats hearts are in a state of insufficient perfusion. Using a 7.0T CMR scanner, we found that the heart structure and function of rats exposed to chronic hypoxia at high altitudes for 12 weeks had undergone some changes [29]. Based on this, we hypothesize that the mechanisms of myocardial injury in cardiovascular diseases at high altitudes are related to the altitude level. Therefore, this study aims to explore the changes in myocardial microcirculation of rats at different altitudes under natural high-altitude conditions using CT myocardial perfusion imaging, combined with blood indicators, pathological tests, qRT-PCR, and Western blot analysis. This approach provides an experimental basis for understanding the mechanisms and prevention of myocardial injury in high-altitude cardiovascular diseases.

## 2 Methods

### 2.1 Study subjects

Sixty 4-week-old SPF-grade male Sprague Dawley (SD) rats were purchased from Sichuan Chengdu Dashuo Experimental Animal Company (Production License No. SCXK (Chuan) 2020-030, Use License No. SYXK (Chuan) 2018-119). This study was approved by the Medical Ethics Committee of Qinghai Provincial People's Hospital and strictly followed the relevant regulations on animal experiments from the Ministry of Health of China.

### 2.2 Grouping and management of experimental animals

The SD rats were transported to different regions with varying altitudes: Chengdu (altitude ∼450 m), Xining (altitude ∼2200 m), Yushu (altitude ∼3800 m), and Maduo (altitude ∼4200 m). These rats were assigned to four groups: the plain group (Chengdu), the moderate-altitude(MA) group (Xining), the high-altitude A(HA-A) group, and the high-altitude B(HA-B) group (Maduo). Each group consisted of 15 rats.

The rats were housed in animal experimental rooms at different altitudes with the following conditions: temperature 18–25 °C, humidity 40–60 %, and normal air circulation with a day-night rhythm matching the outdoor environment. The rats in the Plains, Moderate Altitude, and High Altitude groups were raised until they reached 28 weeks of age.

### 2.3 CT myocardial perfusion imaging (CT-MPI) scanning procedure

#### 2.3.1 Preparation before scanning

Before the scanning, rats were weighed, and anesthesia was induced intraperitoneally with 10 % chloral hydrate (1–2 ml/100 g). After successful anesthesia, the rats were fixed in a prone position and a tail vein puncture was performed using a Y-type needle (0.7 × 19 mm, flow rate 19 ml/min).

#### 2.3.2 CT myocardial perfusion scanning

The scanning personnel wore lead aprons, hats, and neck shields. The rats, fixed on the wooden board, were placed on the CT scanner bed with the chest fully exposed. If signs of awakening were observed during the procedure, additional small doses of anesthetic were administered via the tail vein.

For the CT-MPI scan, a contrast agent (0.2 ml/100 g) was injected via the tail vein, and scanning was started immediately. The rats were swiftly removed from the scanning area and left until the scan was completed, allowing the CT-MPI images to be obtained.

CT Equipment: GE Revolution CT, a high-resolution 256-layer, 128-slice scanner. Scan parameters: tube voltage 80 kV, tube current 100 mA, 16 cm detector width, 5 mm slice thickness, rotation speed 0.5 s/r, small body SFOV, 10 cm DFOV; contrast agent: Iopromide (300 mg/100 ml).

**Fig. 1:**
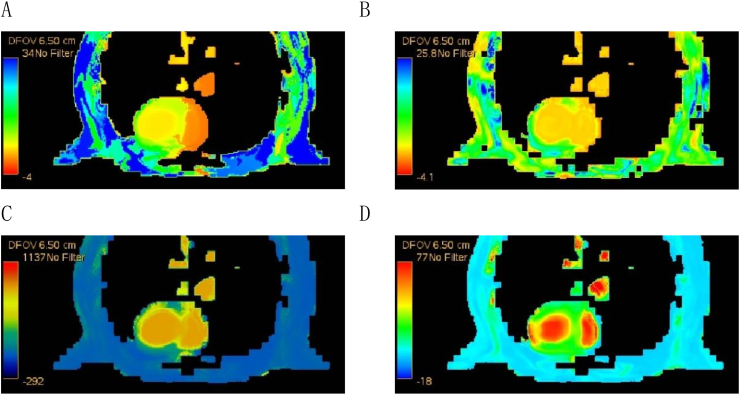
CT myocardial perfusion map of rats in HA-B group. A: TTP diagram; B: MTT diagram; C: BF diagram; D: BV diagram.

The post-processing was conducted using the GE AW4.7 workstation and the 4D perfusion software from the CT tumor body protocol. A myocardial perfusion image was generated, and the left ventricular myocardium was manually outlined in the middle slice using regions of interest (ROI) of 0.1 mm^2^. The average perfusion parameters (TTP, MTT, BF, BV) were automatically calculated (Fig. 1).

### 2.4 Blood collection and pathological sampling

After the CT scan, rats were placed on a surgical table, fixed, and their chest was fully exposed. The aorta was dissected to collect blood into different centrifuge tubes for immediate centrifugation (3000 rpm, 10 min). Blood indicators were measured using a Coulter automated cell counter, including: Red blood cell indicators (RBC, HGB, HCT, MCV, MCH, MCHC, RDW-SD, RDW-CV), White blood cell and platelet indicators (WBC, NEUT#, LYMPH#, MONO#, EO#, BASO#, NEUT%, LYMPH%, MONO%, EO%, BASO%, PLT), and Biochemical indicators (ALT, AST, ALT/AST, TBIL, DBIL, IBIL, GGT, ALP, Cr, LDH, TC).

The heart was harvested by ligating the aortic root, and tissue samples were collected for pathological analysis (HE staining, Masson staining), qRT-PCR, and Western blot assays.

### 2.5 Statistical analysis

Data were analyzed using SPSS 25.0 statistical software. Quantitative data are expressed as mean ± standard deviation ($\bar{x}$ ±s). One-way analysis of variance (ANOVA) was used for inter-group comparisons. The LSD method was applied when variances were homogeneous, and the Tamhane's T2 method was used for heterogeneity of variance. Differences with P < 0.05 were considered statistically significant.

## 3 Results

### 3.1 Myocardial perfusion parameters of rats at different altitudes

**Fig. 2:**
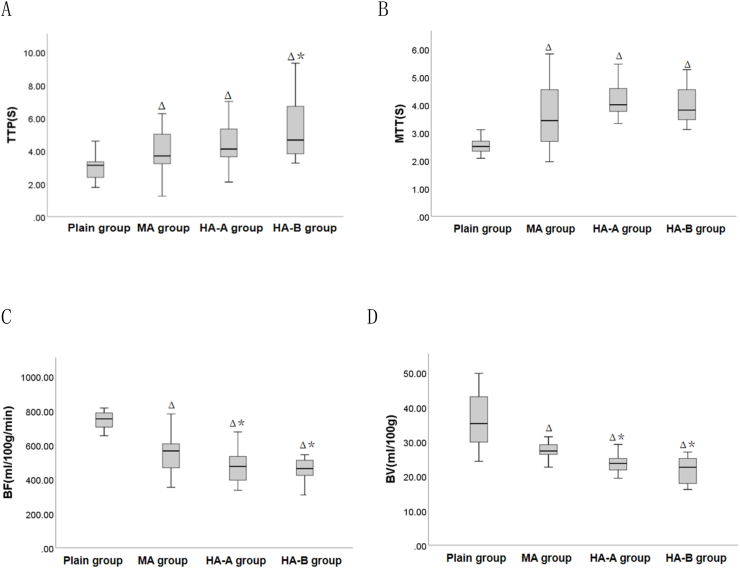
Box plots of myocardial perfusion parameters in plain, MA, HA-A, and HA-B groups. Δ indicates P < 0.05 when compared with the plain group, ∗ indicates P < 0.05 when compared with the MA group, and ^¥^ indicates P < 0.05 when compared with the HA-A group. TTP: time to peak; MTT: mean transit time; BF: blood flow; BV: blood volume.

CT-MPI scan results showed that (Table 1 and Fig. 2), compared to the plain group, the TTP and MTT in the MA, HA-A, and HA-B groups were significantly increased (P < 0.05), while the BF and BV were significantly decreased (P < 0.05). Furthermore, when compared to the MA group, the BF and BV in the HA-A and HA-B groups were significantly decreased (P < 0.05), and the TTP in the HA-B group was significantly increased (P < 0.05). However, no statistical differences were found between HA-A and HA-B groups in terms of TTP, MTT, BF, and BV (P > 0.05).

**Table 1:** Comparison of myocardial perfusion parameters among plain group, MA group, HA-A group and HA-B group($\bar{x}$ ±s).

Index	plain group(n = 15)	MA group(n = 15)	HA-A group(n = 14)	HA-B group(n = 15)	F value	*P* value
TTP(s)	2.93 ± 0.81	4.03 ± 1.37^Δ^	4.33 ± 1.37^Δ^	5.30 ± 1.88^Δ∗^	7.197	<0.001
MTT(s)	2.61 ± 0.45	3.59 ± 1.14^Δ^	4.22 ± 0.72^Δ^	4.01 ± 0.67^Δ^	12.262	<0.001
BF(ml/100 g/min)	725.15 ± 93.63	561.33 ± 125.82^Δ^	470.68 ± 97.66^Δ∗^	460.40 ± 96.13^Δ∗^	20.508	<0.001
BV(ml/100 g)	36.50 ± 8.27	28.04 ± 3.48^Δ^	24.22 ± 3.55^Δ∗^	21.75 ± 3.99^Δ∗^	22.516	<0.001

Note: ^Δ^ indicates P < 0.05 when compared with the plain group, ∗ indicates P < 0.05 when compared with the MA group, and ^¥^ indicates P < 0.05 when compared with the HA-A group. TTP: time to peak; MTT: mean transit time; BF: blood flow; BV: blood volume.

### 3.2 Blood parameters of rats at different altitudes

#### 3.2.1 Red blood cell parameters

**Fig. 3:**
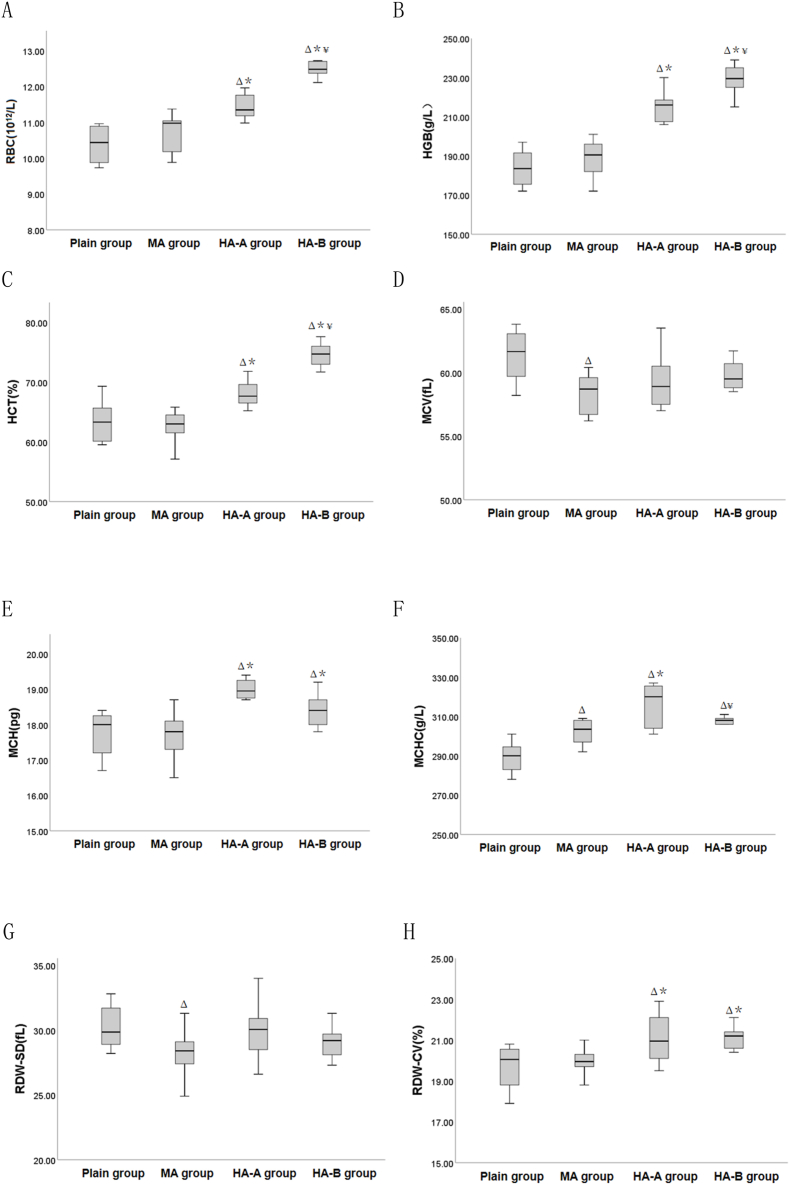
Box plots of red blood cell indexes among plain, MA, HA-A, and HA-B groups. ^Δ^ indicates P < 0.05 when compared with the plain group, ∗ indicates P < 0.05 when compared with the MA group, and ^¥^ indicates P < 0.05 when compared with the HA-A group.

Compared to the plain group (Table 2 and Fig. 3), the MCHC in the MA group was significantly increased (P < 0.05), while the MCV and RDW-SD were significantly decreased (P < 0.05). In the HA-A and HA-B groups, RBC, HGB, HCT, MCH, MCHC, and RDW-CV were significantly increased (P < 0.05). Compared to the MA group, RBC, HGB, HCT, MCH, MCHC, and RDW-CV were significantly increased in both the HA-A and HA-B groups (P < 0.05). Among the high-altitude groups, the RBC, HGB, and HCT were significantly higher in the HA-B group than in the HA-A group (P < 0.05), while the MCHC was significantly lower (P < 0.05).

**Table 2:** Comparison of red blood cell indexes among plain group, MA group, HA-A group and HA-B group($\bar{x}$ ±s).

Index	plain group(n = 8)	MA group(n = 10)	HA-A group(n = 8)	HA-B group(n = 10)	Fvalue	*P* value
RBC(10 [12]/L)	10.04 ± 1.33	10.71 ± 0.53	11.44 ± 0.36^Δ∗^	12.44 ± 0.53^Δ∗¥^	17.010	<0.001
HGB(g/L)	177.80 ± 22.79	188.70 ± 9.33	215.00 ± 8.00^Δ∗^	229.10 ± 7.30^Δ∗¥^	30.027	<0.001
HCT(%)	61.45 ± 7.67	62.46 ± 2.95	68.06 ± 2.25^Δ∗^	74.65 ± 2.07^Δ∗¥^	19.765	<0.001
MCV(fL)	61.35 ± 2.04	55.37 ± 10.20^Δ^	59.29 ± 2.24	60.04 ± 1.69	1.942	0.143
MCH(pg)	17.75 ± 0.67	17.64 ± 0.70	18.83 ± 0.67^Δ∗^	18.44 ± 0.44^Δ∗^	7.173	0.001
MCHC(g/L)	289.25 ± 7.74	302.10 ± 6.15^Δ^	315.88 ± 11.22^Δ∗^	306.90 ± 3.96^Δ¥^	17.861	<0.001
RDW-SD(fL)	30.24 ± 1.66	28.30 ± 1.66^Δ^	29.94 ± 2.21	29.07 ± 1.26	2.401	0.086
RDW-CV(%)	19.69 ± 1.10	19.96 ± 0.66	21.09 ± 1.25^Δ∗^	21.02 ± 0.80^Δ∗^	4.957	0.006

Note: ^Δ^ indicates P < 0.05 when compared with the plain group, ∗ indicates P < 0.05 when compared with the MA group, and ^¥^ indicates P < 0.05 when compared with the HA-A group.

#### 3.2.2 White blood cell and platelet parameters

**Fig. 4:**
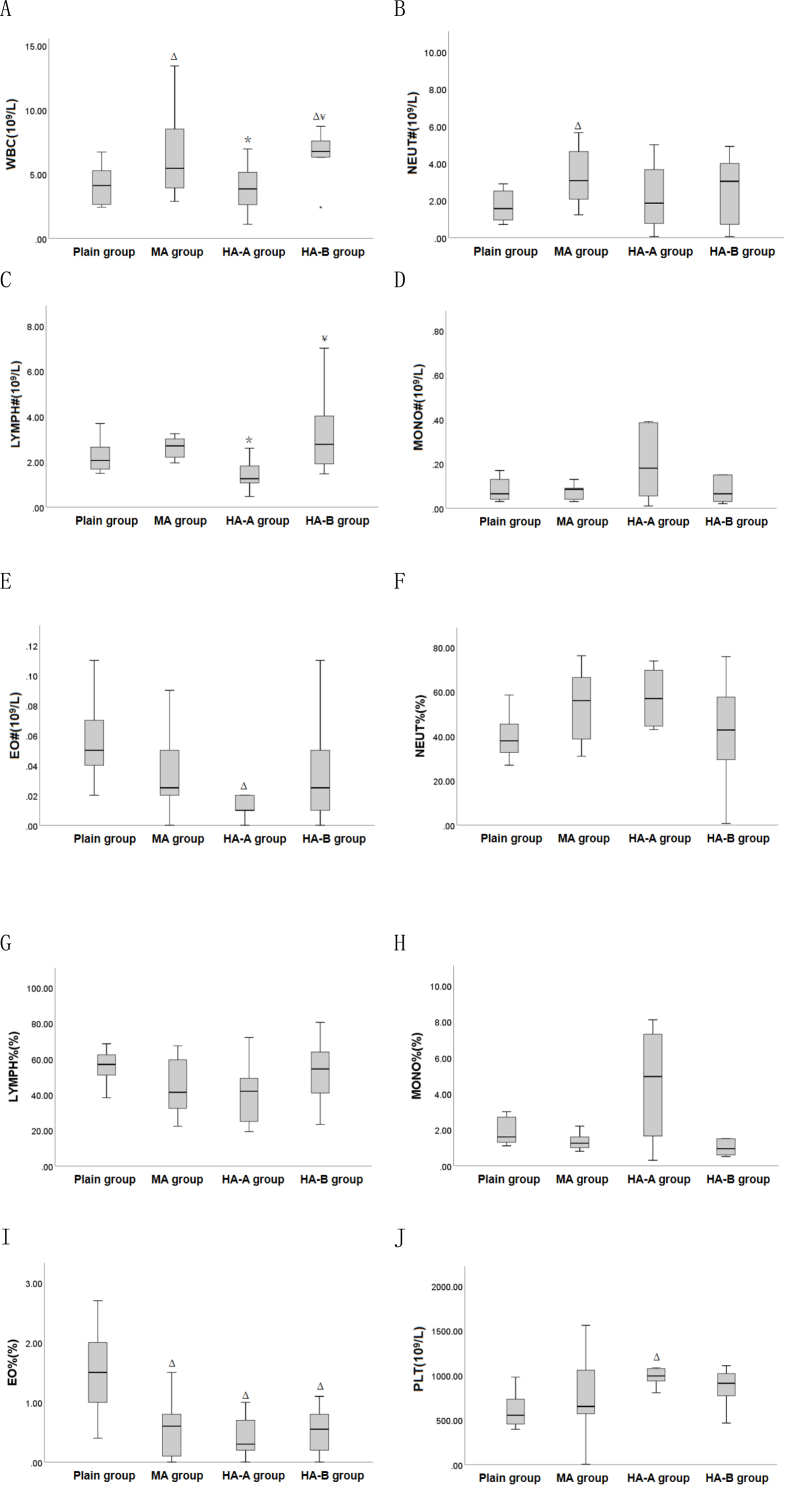
Box plots of white blood cell and platelet indexes indexes among plain, MA, HA-A, and HA-B groups. ^Δ^ indicates P < 0.05 when compared with the plain group, ∗ indicates P < 0.05 when compared with the MA group, and ^¥^ indicates P < 0.05 when compared with the HA-A group.

Compared to the plain group (Table 3 and Fig. 4), WBC and NEUT# in the MA group were significantly increased (P < 0.05), while EO% was significantly decreased (P < 0.05). In the HA-A group, EO# and EO% were significantly decreased (P < 0.05), while PLT was significantly increased (P < 0.05). In the HA-B group, WBC was significantly increased (P < 0.05), and EO% was significantly decreased (P < 0.05). Compared to the MA group, WBC and LYMPH# were significantly decreased in the HA-A group (P < 0.05), while WBC and LYMPH# were significantly increased in the HA-B group (P < 0.05).

**Table 3:** Comparison of white blood cell and platelet indexes among plain group, MA group, HA-A group and HA-B group($\bar{x}$ ±s).

Index	plain group(n = 8)	MA group(n = 10)	HA-A group(n = 8)	HA-B group(n = 10)	Fvalue	*P* value
WBC(10 [9]/L)	4.15 ± 1.56	6.54 ± 3.26^Δ^	3.91 ± 1.91^∗^	6.59 ± 2.25^Δ¥^	3.325	0.032
NEUT#(10 [9]/L)	1.70 ± 0.86	3.68 ± 2.66^Δ^	2.20 ± 1.78	2.54 ± 1.85	1.696	0.187
LYMPH#(10 [9]/L)	2.24 ± 0.74	2.72 ± 0.74	1.42 ± 0.66^∗^	3.42 ± 1.91^¥^	3.785	0.020
MONO#(10 [9]/L)	0.15 ± 0.21	0.10 ± 0.08	0.28 ± 0.31	0.59 ± 1.24	1.057	0.381
EO#(10 [9]/L)	0.06 ± 0.03	0.04 ± 0.03	0.01 ± 0.01^Δ^	0.03 ± 0.03	3.483	0.027
BASO#(10 [9]/L)	0.01 ± 0.01	0.01 ± 0.01	0.01 ± 0.01	0.01 ± 0.00	0.072	0.975
NEUT%(%)	39.67 ± 10.16	53.52 ± 16.14	52.16 ± 23.41	39.18 ± 24.65	1.427	0.253
LYMPH%(%)	55.90 ± 9.63	44.47 ± 16.03	40.43 ± 17.31	51.82 ± 17.20	1.696	0.187
MONO%(%)	2.76 ± 2.92	1.34 ± 0.42	6.84 ± 8.43	8.38 ± 18.27	0.941	0.432
EO%(%)	1.51 ± 0.73	0.59 ± 0.47^Δ^	0.43 ± 0.35^Δ^	0.51 ± 0.38^Δ^	8.577	<0.001
BASO%(%)	0.16 ± 0.17	0.08 ± 0.08	0.15 ± 0.17	0.11 ± 0.10	0.755	0.528
PLT(10 [9]/L)	606.75 ± 196.27	749.22 ± 445.94	1024.75 ± 172.76^Δ^	853.80 ± 223.89	2.963	0.047

Note: ^Δ^ indicates P < 0.05 when compared with the plain group, ∗ indicates P < 0.05 when compared with the MA group, and ^¥^ indicates P < 0.05 when compared with the HA-A group.

#### 3.2.3 Blood biochemical parameters

**Fig. 5:**
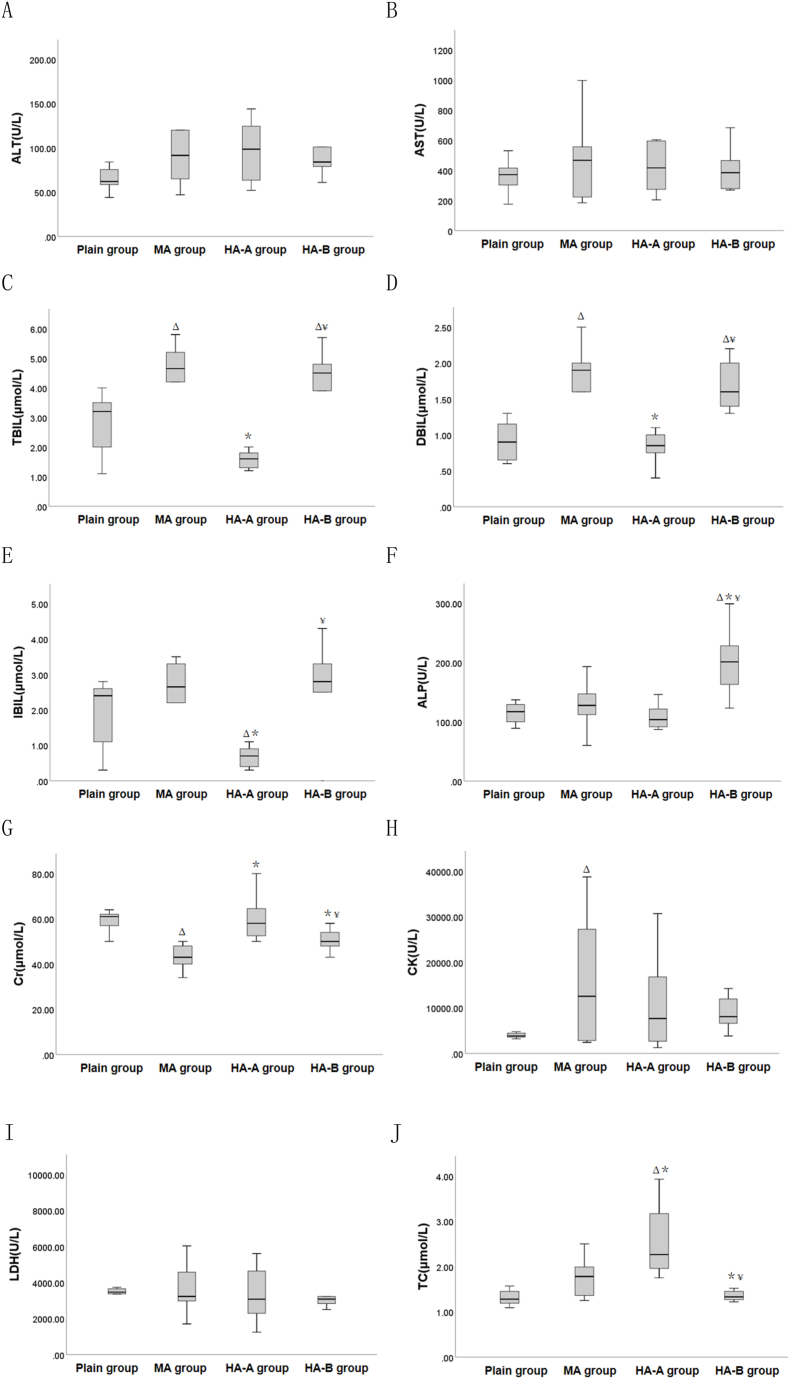
Box plots of blood biochemical indexes among plain, MA, HA-A, and HA-B groups. ^Δ^ indicates P < 0.05 when compared with the plain group, ∗ indicates P < 0.05 when compared with the MA group, and ^¥^ indicates P < 0.05 when compared with the HA-A group.

Compared to the plain group (Table 4 and Fig. 5), TBIL, DBIL, GGT, and CK in the MA group were significantly increased (P < 0.05), while Cr was significantly decreased (P < 0.05). In the HA-A group, IBIL was significantly decreased (P < 0.05), while TC was significantly increased (P < 0.05). In the HA-B group, TBIL, DBIL, GGT, and ALP were significantly increased (P < 0.05). Compared to the MA group, TBIL, DBIL, IBIL, GGT were significantly decreased in the HA-A group (P < 0.05), while Cr and TC were significantly increased (P < 0.05). In the HA-B group, GGT, ALP, and Cr were significantly increased (P < 0.05), while TC was significantly decreased (P < 0.05).

**Table 4:** Comparison of blood biochemical indexes among plain group, MA group, HA-A group and HA-B group($\bar{x}$ ±s).

Index	plain group(n = 7)	MA group(n = 10)	HA-A group(n = 8)	HA-B group(n = 9)	Fvalue	*P* value
ALT(U/L)	70.00 ± 23.54	117.40 ± 77.20	114.75 ± 77.98	89.67 ± 21.40	1.165	0.339
AST(U/L)	358.71 ± 113.68	493.90 ± 285.05	490.00 ± 309.98	405.67 ± 135.27	0.650	0.589
ALT/AST	0.23 ± 0.05	0.24 ± 0.05	0.25 ± 0.08	0.23 ± 0.07	0.170	0.916
TBIL(μmol/L)	2.76 ± 1.09	4.29 ± 1.38^Δ^	1.58 ± 0.29^∗^	4.09 ± 1.46^Δ¥^	9.859	<0.001
DBIL(μmol/L)	0.91 ± 0.29	1.73 ± 0.64^Δ^	0.90 ± 0.35^∗^	1.59 ± 0.59^Δ¥^	6.209	0.002
IBIL(μmol/L)	1.81 ± 1.05	2.56 ± 0.92	0.68 ± 0.29^Δ∗^	2.50 ± 1.47^¥^	6.088	0.002
GGT(U/L)	1.00 ± 0.00	2.50 ± 0.97^Δ^	0.75 ± 0.46^∗^	3.67 ± 1.87^Δ∗¥^	12.299	<0.001
ALP(U/L)	127.86 ± 48.27	130.80 ± 40.29	108.25 ± 20.42	200.33 ± 56.10^Δ∗¥^	7.469	0.001
Cr(μmol/L)	59.00 ± 5.29	43.20 ± 5.33^Δ^	60.00 ± 9.83^∗^	52.00 ± 7.65^∗¥^	10.277	<0.001
CK(U/L)	3952.14 ± 589.28	15415.50 ± 12969.81^Δ^	10764.13 ± 10466.29	10930.33 ± 8075.09	1.931	0.146
LDH(U/L)	3478.86 ± 870.85	4046.80 ± 1920.30	3356.63 ± 1508.16	3031.22 ± 753.06	0.882	0.461
TC(μmol/L)	1.32 ± 0.19	1.76 ± 0.41	2.56 ± 0.78^Δ∗^	1.31 ± 0.20^∗¥^	12.992	<0.001

Note: ^Δ^ indicates P < 0.05 when compared with the plain group, ∗ indicates P < 0.05 when compared with the MA group, and ^¥^ indicates P < 0.05 when compared with the HA-A group.

### 3.3 Histological HE staining of myocardium in rats at different altitudes

**Fig. 6:**
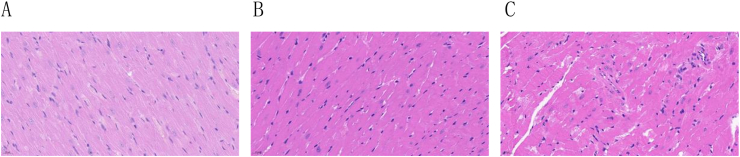
HE staining of myocardial tissue of rats at different altitudes (400 × ). A: Plain group; B: MA group; C: HA-B group.

HE staining results showed that the myocardial structure of rats in the plain group was clear, with orderly myocardial fiber arrangement. However, with increasing altitude, the myocardial fiber arrangement in the MA and HA-B groups became increasingly disordered, with enlarged cell volumes, widened intercellular spaces, and infiltration of inflammatory and fat cells in the interstitial space (Fig. 6).

### 3.4 Histological masson staining of myocardium in rats at different altitudes

**Fig. 7:**
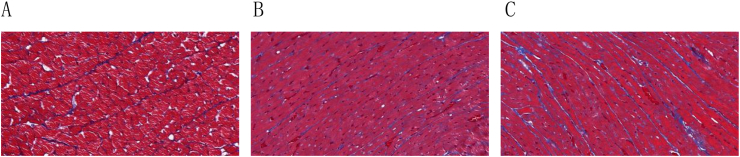
Masson staining of rat myocardial tissue at different altitudes (400 × ). A: Plain group; B: MA group; C: HA-B group (400 × ).

**Fig. 8:**
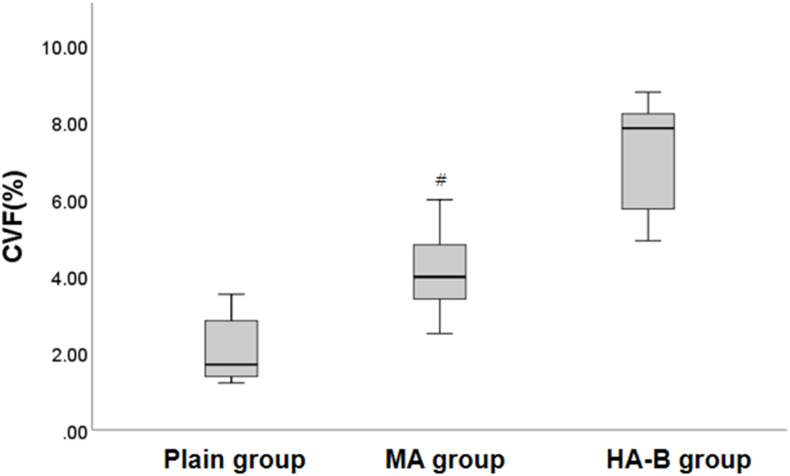
Box plot of collagen volume fraction (CVF) in rat myocardial tissue at different altitudes. ^Δ^ indicates P < 0.05 when compared with the plain group, ∗ indicates P < 0.05 when compared with the MA group.

Masson staining revealed that in the plain group, myocardial cells were arranged orderly and collagen fibers in the myocardial tissue were clearly stained with moderate distribution. With the increase in altitude, the arrangement of myocardial fibers became increasingly disordered in the MA and HA-B groups, with increasing collagen fibers in the myocardial interstitial spaces and around blood vessels, showing signs of myocardial remodeling. Image-J software analysis showed that the collagen volume fraction (CVF) in the MA and HA-B groups was significantly increased compared to the plain group (P < 0.05), and the CVF in the HA-B group was significantly greater than in the MA group (P < 0.05) (Table 5, Fig. 7, Fig. 8).

**Table 5:** Comparison of myocardial CVF among plain group, MA group and HA-B group ($\bar{x}$ ±s).

Index	plain group(n = 5)	MA group(n = 7)	HA-B group(n = 6)	F value	*P* value
CVF(%)	2.13 ± 1.00	4.13 ± 1.19^#^	7.23 ± 1.54^#^∗	22.589	<0.001

Note: ^Δ^ indicates P < 0.05 when compared with the plain group, ∗ indicates P < 0.05 when compared with the MA group.

### 3.5 qRT-PCR detection of myocardial tissue in rats at different altitudes

**Fig. 9:**
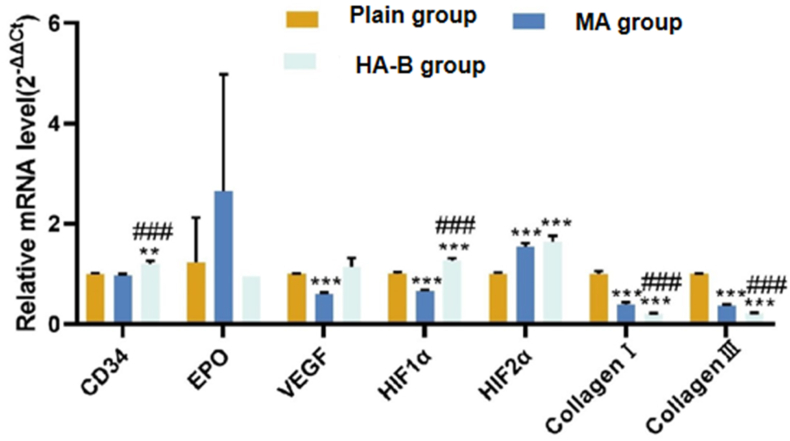
mRNA expression levels of CD34, EPO, VEGF, HIF-1α, HIF-2α, collagen type I, and collagen type III detected by qRT-PCR in myocardial tissue of plain group, MA group, and HA-B group. Compared with plain group, ∗∗P < 0.01, ∗∗∗P < 0.001; Compared with the MA group, ^###^P < 0.001.

qRT-PCR results showed that (Fig. 9), compared to the plain group, the mRNA expression of HIF-2α in the MA group was significantly increased (P < 0.05), while the mRNA expression of VEGF, HIF-1α, Type I collagen, and Type III collagen was significantly decreased (P < 0.05). In the HA-B group, the mRNA expression of CD34, HIF-1α, and HIF-2α was increased (P < 0.05), while the mRNA expression of Type I and Type III collagen was decreased (P < 0.05). Compared to the MA group, the mRNA expression of CD34 and HIF-1α was significantly increased in the HA-B group (P < 0.05), while the mRNA expression of Type I and Type III collagen was significantly decreased (P < 0.05).

### 3.6 Western blot detection of myocardial tissue proteins in rats at different altitudes

**Fig. 10:**
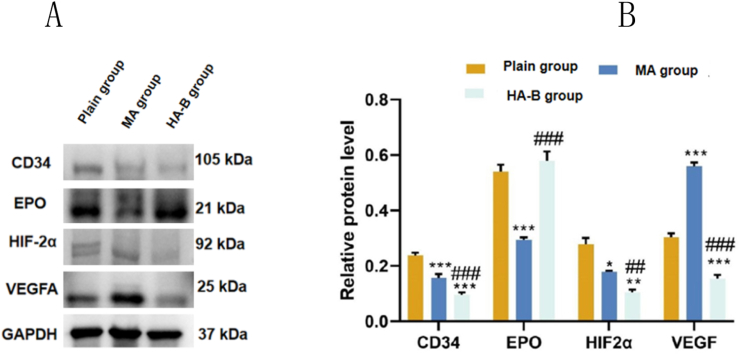
Protein expression levels of CD34, EPO, HIF-2α and VEGF in myocardial tissue of plain group, MA group and HA-B group were detected by Western blot. Compared with plain group, ∗P < 0.05, ∗∗P < 0.01, ∗∗∗P < 0.001; Compared with the MA group, ^##^P < 0.01, ^###^P < 0.001.

Western blot results showed that (Fig. 10), compared to the plain group, the protein expression of VEGF was significantly increased in the MA group (P < 0.05), while the protein expressions of CD34, EPO, and HIF-2α were significantly decreased (P < 0.05). In the HA-B group, the protein expression of EPO was significantly increased (P < 0.05), while the protein expressions of CD34, HIF-2α, and VEGF were significantly decreased (P < 0.05). Compared to the MA group, the protein expression of EPO was significantly increased in the HA-B group (P < 0.05), while the protein expressions of CD34, HIF-2α, and VEGF were significantly decreased (P < 0.05).

## 4 Discussion

The unique environmental characteristics of high-altitude regions, such as low atmospheric pressure and low oxygen levels, have a significant impact on multiple systems of the human body, particularly the cardiovascular system [6]. These environmental factors can lead to changes in heart structure and function. Studies have shown that populations migrating to or residing at high altitudes are prone to developing acute or chronic high-altitude diseases, though the exact mechanisms of these diseases are not yet fully understood. In recent years, with deeper clinical and basic research into cardiovascular diseases, myocardial microcirculation has become a hot topic, and clinicians have increasingly recognized its importance in cardiovascular health. However, research on myocardial microcirculation in acute and chronic high-altitude diseases remains relatively limited.

With the rapid development of medical imaging devices, particularly CT myocardial perfusion imaging (CT-MPI), this technology has matured to the point where it not only accurately provides anatomical information about coronary arteries and the heart but also evaluates myocardial perfusion, offering significant advantages for assessing myocardial microcirculation in a combined anatomical and functional context. Our previous research using CT-MPI found that under the natural low-pressure and low-oxygen environment of high altitudes, SD rats' hearts were in a state of insufficient perfusion, and right ventricular function and pathology had changed [20 21]. This suggested that the mechanism of cardiovascular damage at high altitudes might be related to the elevation. Building on this, the current study further investigates changes in myocardial microcirculation in rats at different altitudes. As shown in Table 1 and Fig. 2, with increasing altitude, significant changes were observed in the myocardial perfusion parameters of the rats: TTP and MTT gradually prolonged, while BF and BV gradually decreased. In high-altitude rats, BF and BV were the first to decrease, followed by a significant increase in TTP with increasing altitude. These results further support our hypothesis that as altitude increases, myocardial microcirculation changes, and the heart enters a state of low perfusion. The physiological mechanisms behind these changes could be due to the decreasing atmospheric pressure and oxygen content. The reduced oxygen content stimulates the expression of HIF-1α, which in turn activates the expression of vascular endothelial growth factor (VEGF), promoting angiogenesis and endothelial cell proliferation, increasing vascular permeability. These changes could lead to an increase in capillaries in myocardial microcirculation, extending the time blood flows through these capillaries, resulting in prolonged TTP and MTT. Meanwhile, the decreased blood flow and vascular volume could be caused by an increase in red blood cell count and blood viscosity, which slow the blood flow, further contributing to myocardial perfusion deficiency.

Previous studies have explored the relationship between blood routine and biochemical indicators in populations living at high altitudes, identifying an increased percentage of eosinophils (EO%) and hemoglobin (HGB) as major risk factors for chronic mountain sickness (CMS) [30]. Our study also found that at different altitudes, changes in red blood cell indices, white blood cell indices, platelet indices, and biochemical parameters were significant [31]. Compared to the plains group, rats in the moderate and high-altitude regions showed notable increases in red blood cell indices (such as RBC, HGB, and HCT), while white blood cell and platelet indices (such as WBC, PLT) also showed marked increases or changes. Moreover, biochemical parameters like TBIL, DBIL, GGT were significantly altered. These changes in blood indices could be attributed to the body's response to the low-oxygen environment by increasing red blood cell count and hemoglobin concentration to enhance oxygen-carrying capacity, thereby maintaining homeostasis under the high-altitude conditions.

Previous studies have reported that high-altitude low-pressure hypoxic conditions can induce myocardial tissue remodeling, leading to myocardial hypertrophy and fibrosis [[32], [33], [34]]. Histopathological analysis further revealed significant changes in myocardial tissue structure with increasing altitude. HE and Masson staining showed that myocardial fibers in the rats became increasingly disorganized as altitude increased. The intercellular spaces widened, myocardial cell volumes enlarged, and infiltrates of inflammatory cells and adipocytes appeared. These pathological changes suggest that the myocardial tissue underwent remodeling and fibrosis in response to the high-altitude low-oxygen environment.

At the molecular level, qRT-PCR analysis indicated a significant upregulation of genes related to hypoxia, including HIF-1α, HIF-2α, and VEGF, as altitude increased. This suggests that the body adapts to the hypoxic environment by regulating these molecular pathways to promote changes in myocardial microcirculation. Moreover, qRT-PCR results indeed showed that HIF-2α mRNA expression was elevated at moderate altitude compared to plain areas, and its expression, along with that of CD34 and HIF-1α, increased further with higher altitudes. This pattern led us to hypothesize that HIF-2α is more sensitive to changes in altitude. This provides new insights into understanding the mechanisms of high-altitude cardiovascular diseases.

Western blot analysis of myocardial tissues from the plain and HA-B group rats revealed that as altitude increased, the protein expression of VEGF and EPO first increased, followed by EPO at higher altitudes. These findings further suggest that the protein expression of CD34, EPO, HIF-2α, and VEGF in myocardial tissue changes with altitude, leading to alterations in myocardial microcirculation.

In conclusion, the results of this study show that myocardial microcirculation in rats from different altitudes undergoes significant changes, with the heart entering a state of low perfusion. TTP and MTT gradually prolonged, while BF and BV gradually decreased. In high-altitude rats, BF and BV were the first to decrease, followed by a significant increase in TTP as altitude rose. Combining blood indices, histopathology (HE and Masson staining), qRT-PCR, and Western blot analysis, we found changes in various blood indices, myocardial tissue structure remodeling, and the expression of genes and proteins involved in myocardial microcirculation. We speculate that HIF-2α is more sensitive to changes in altitude. The findings provide experimental evidence for the changes in myocardial microcirculation under high-altitude low-pressure and low-oxygen conditions, and also lay a solid foundation for further research on high-altitude cardiovascular diseases.

## Ethics approval and consent to participate

This study was approved by the Medical Ethics Committee of Qinghai Provincial People's Hospital (2020-115).

## Consent for publication

All the authors listed have approved the manuscript to be published.

## Author contributions

Yanqiu Sun and Chunlong Yan conceived and designed the study. Jinfeng Ma, Tingjun Yan, Dengfeng Tian and Chenhong Zhang contributed to data collection. Chunlong Yan contributed to the writing of the report. All authors read and approved the final manuscript.

## Funding

The work was supported by the Qinghai province “Kunlun Elite High-end Innovative and Entrepreneurial Talents” Program To Cultivate Leading Talents (Project No. Youth Talent Word (2021) No. 13),the Key R&D Program of Jining(No. 2023YXNS103) Sponsored by PhD ResearchFund of Jining NO.1 Peoples Hospital (2025-BS-005), and the Beijing Postdoctoral Research Foundation.

## Declaration of competing interest

The authors declare that they have no competing interests.

## Data Availability

Data will be made available on request.

## Abbreviations

CT-MPI = computed tomography myocardial perfusion imaging, CAG = coronary angiography, CMD = coronary microvascular dysfunction, CMVD = coronary microvascular disease, TTDE = transthoracic Doppler echocardiography, MCE = myocardial contrast echocardiography, CT = computed tomography, PET = positron emission tomography, SPECT = single photon emission computed tomography, CMRI = cardiac magnetic resonance imaging, CCTA=Coronary computed tomography angiography, TTP = time to peak, MTT = mean transit time, BF = blood flow, BV = blood volume, CMS = chronic mountain sickness, RBC=Red blood cell count, HGB=Hemoglobin, HCT=Hematocrit, MCV = mean corpuscular volume, MCH = mean corpuscular hemoglobin, MCHC = mean corpuscular hemoglobin concentration, RDW = red blood cell volume distribution width, WBC=White blood cell count, NEUT=Neutrophil, LYMPH = Lymphocytes, MONO=Monocytes,EO = Eosinophils, NEUT% = Percentage of neutrophils, LYMPH% = Percentage of lymphocytes, MONO% = Percentage of monocytes,EO% = Percentage of eosinophils,PLT=Platelet count,ALT = Alanine aminotransferase, AST = Aspartate amino transferase, TBIL = Total bilirubin, DBIL = Direct bilirubin, IBIL=Indirect bilirubin, GGT = Gamma-glutamyltransferase, ALP=Alkaline phosphatase, Cr = creatinine, CK=Creatine kinase, LDH=Lactate dehydrogenase, TC = Total cholesterol.
